# Relationship between Nomophobia, Various Emotional Difficulties, and Distress Factors among Students

**DOI:** 10.3390/ejihpe12070053

**Published:** 2022-07-05

**Authors:** Lea Santl, Lovorka Brajkovic, Vanja Kopilaš

**Affiliations:** 1 Department of Psychology, Faculty of Croatian Studies, University of Zagreb, Borongajska 83 D, 10000 Zagreb, Croatia; lsantl@hrstud.hr (L.S.); lbrajkov1@hrstud.hr (L.B.); 2 Croatian Institute for Brain Research, School of Medicine, University of Zagreb, Salata 12, 10000 Zagreb, Croatia

**Keywords:** nomophobia, emotional skills and competencies, loneliness, distress factors

## Abstract

The concept of nomophobia is a relatively new and is defined as the fear of not being able to use all the features and benefits provided by smartphones. The main goal of this research was to examine the relationship between nomophobia and various emotional difficulties and distress factors. The following measuring instruments were used on a sample of 257 Croatian students whose average age was 22 years: Nomophobia Questionnaire (NMP-Q); Scales of Depression, Anxiety, and Stress (DASS); Social and Emotional Loneliness Scale (SELSA); and Emotional Skills and Competences Questionnaire (ESCQ-15). Significant correlations between nomophobia and all examined variables were determined. It was found that nomophobia and emotional skills and competence act as significant predictors in expression of distress factors: 30% of the variance in the severity of depression symptoms, 24% of the variance in the severity of anxiety symptoms, and 26% of the variance in the severity of stress symptoms were explained.

## 1. Introduction

In the last few years, smartphones have become one of the main parts of a person’s everyday life. Thanks to the range of applications and benefits they provide, these smart devices have become an indispensable aid in business, learning, entertainment, and everyday functioning. In addition, smartphones can also serve as a way of coping with stressful situations by providing a range of different contents [1]. Although smartphones significantly facilitate people’s daily lives, studies on negative correlations and consequences of excessive use of smartphones are worrying. Accordingly, Pera [2] stated that excessive use of these devices or their problematic use can leave negative consequences on the human brain and related psychological processes. He further explained that the problematic use of mobile phones was negatively linked to the psychosocial well-being of young people and affected their mental health. Some research confirmed that the use of smartphones, which can be considered problematic, is associated with the severity of depressive symptomatology and anxiety symptoms, lower self-esteem, perceived social support, and emotional dysregulation. Due to the growing involvement of smartphones in human life and their impact on various psychological processes, a relatively new phenomenon that is associated with smart devices, called nomophobia, has surfaced. It is a type of behavioural addiction to smartphones based on feelings of anxiety and restlessness or anxiety in situations where a person is unable to access a smart device and reach all the benefits it provides [3]. Yildirim and Correia [4] offered another definition of nomophobia as a fear of not being able to use smartphones and the various services they provide.

On the other hand, nomophobia can also be observed in terms of addiction, such as internet addiction or smartphone addiction. Proponents of this approach often define nomophobia as an addiction to online social networks, and they explain how people with intense levels of nomophobia perceive a smart device as a source of comfort and feelings of relief (like other addictive substances) [5].

Considering previous theoretical knowledge and definitions of nomophobia, nomophobia can be viewed as a disorder characterized by behaviours with different clinical characteristics; it can cause several psychological symptoms and implies a pathological fear that a person will not be able connect with new technologies [6]. In addition, nomophobia can be defined in terms of behavioural dependence but can also be viewed as situational phobia.

It is quite clear that smartphones are becoming more popular every day because of the many benefits they provide. According to the latest data, there are currently 6.37 billion smartphone users in the world, and that number will increase over the years [7]. Accordingly, it is logical to assume that the prevalence of nomophobia will increase with time as well, as it is based on the excessive use of smartphones. In a study among participants whose ages ranged from 17 to 29 years, the authors found that 71.5% of participants exhibited moderate levels of nomophobia and 8.5% severe symptoms of nomophobic behaviours [8]. Bodrožić Selak conducted the first research of Croatian population on this topic and concluded that students show moderate levels of nomophobia [9]. Gržan confirmed a slight increase in the severity of symptoms of nomophobia in a sample of Croatian students [10].

### 1.1. Depression and Nomophobia

Young and Rogers found that more pronounced depressive symptomatology was associated with excessive Internet use [11]. It is possible that the basis of this connection is characteristics that characterize a depressed person, such as low self-esteem, lack of motivation, and the need for approval, which contribute to more frequent online communication and excessive use of the Internet. Similar findings were confirmed in other study, where a positive relationship between problematic smartphone use and depression was found [12]. In one of a few studies on this topic, Sharma et al. [13] found a significant positive relationship of nomophobia with depression. The same authors explain that there is a possibility that adolescents who express depressive symptoms more use social networks to reduce their levels of loneliness and to improve their self-esteem.

### 1.2. Anxiety and Nomophobia

King et al. stated that participants diagnosed with panic disorder in situations without access to a smartphone experience more intense mental and physical symptoms [14]. It is possible that the use of mobile phones has a positive effect on people with panic disorder and alleviates the symptoms of a panic attack. Another study found that individuals who reported higher levels of anxiety were more likely to resort to online forms of communication to alleviate their own anxiety symptoms [15].

Ayar et al., in their study, tried to determine the relationship between nomophobia and type of social anxiety connected with body image, and they confirmed that people who experience higher levels of anxiety because of their body image and self-perception of their body achieve higher results on the nomophobia scale [16].

Bekaroğlu and Yılmaz stated that there is a correlation between nomophobia and symptoms of various negative psychological states such as anxiety and depression [17]. More specifically, it is possible that the fear of not being able to use smartphones intensifies the symptoms of stress, anxiety, and depression, but it is possible that individuals with the aforementioned symptoms are more susceptible to developing nomophobia.

### 1.3. Stress and Nomophobia

Individuals who showed nomophobic behaviours experience more intense levels of stress in those situations where they cannot access a mobile phone [18]. The connection between nomophobia and stress can also be explained by the fact that smartphones often provide a way of coping with difficulties. Nomophobia can be a mediator between different behaviours and habits associated with smartphone use and stress, anxiety, and depression. More specifically, individuals who spend more time searching social media experience higher levels of nomophobia and then more severe symptoms of the distress factors [9]. The connection between nomophobia and stress can also be explained by the fact that smartphones often act as a means of coping with difficulties. When a person experiences stress, he/she often reaches for a smartphone and all the benefits it provides, and this can lead to excessive and problematic use of these modern forms of technology, which are at the root of nomophobia [19].

### 1.4. Loneliness and Nomophobia

Research conducted on a sample of adolescents found a positive relationship between nomophobia and loneliness in which nomophobia was a significant predictor of loneliness [20]. Furthermore, the same authors explain that individuals who do not have access to their own smartphone experience a feeling of loneliness, which is based on fear of not being able to communicate and socialize with others [20]. Yildiz Durak also found that there is a positive relationship between nomophobia and loneliness and added that people who have difficulty communicating one-on-one are more likely to resort to online communication, which can remove them from the real world, and as result, loneliness occurs [21]. This can then be manifest in the form of negative emotions, which can result in distance from the social environment and reduced motivation. One research found that those adolescents who spend more time behind the screen of smartphones experience higher levels of anxiety and loneliness and consequently show higher levels of nomophobia [22].

### 1.5. Emotional Skills and Nomophobia

It can be assumed that individuals whose smartphone use can be characterized as problematic experience more serious psychological problems precisely because of poor, maladaptive strategies of emotional regulation [10]. Engelberg and Sjoberg found that Internet addicts are worse at recognizing and decoding emotions [23]. In addition, individuals who have difficulty coping with negative emotional states often turn to the Internet and social media, which can potentially lead to addictive behaviours and ultimately result in pronounced levels of nomophobia [24].

### 1.6. Purpose of the Study

Based on the previous literature and research, there is undeniable relationship between nomophobia and everyday functioning. As nomophobia is relatively new dependency, there are many things that remain unclear about the mechanisms behind it. Hence, the main goal of this research is to examine the relationship between nomophobia, various emotional skills and competence, emotional difficulties (social and emotional loneliness), and distress factors (depression, anxiety, stress).

We hypothesize that there will be positive correlations between factors of distress (anxiety, depression, and stress) and level of nomophobia; there will be a positive relationship between level of nomophobia and emotional and social loneliness and a negative relationship between nomophobia and emotional skills; and we assumed that nomophobia will be a positive predictor of distress factors (anxiety, depression, stress).

## 2. Methods

### 2.1. Participants and Procedure

The research was conducted in the period from 20 December 2021 to 20 January 2022 via the Internet, and the target sample consisted of students of one university in Croatia. The research was anonymous and voluntary. At the beginning, all participants were initially introduced to the purpose of the research; confidentiality and personal data protection were guaranteed, and they were assured that participation in the research was completely voluntary, and they could drop out at any time and that their identity could not be determined in any way. If they decided to take part in this research, they were asked to check the box at the beginning of the survey: “I consent, begin the study”. Ethical approval was obtained from the Department of Psychology, Faculty of Croatian Studies, University of Zagreb (protocol number: 640-16/21-2/0005).

A total of 257 students participated in the research, of which 36 were male students (14%) and 222 female students (86%). The age of the participants ranged from 18 to 42 years of age (M = 22.25, SD = 2.63).

### 2.2. Measures

The survey consisted of the questionnaire made for the purpose of this study, which collected information about participants’ sociodemographic info and smartphone use. In the other part of the survey, participants were asked to answer questions from already validated measures in the Croatian language that investigate nomophobia, depression, anxiety, stress, loneliness, emotional skills, and competences.

Sociodemographic questionnaire and smartphone use.

At the beginning of the questionnaire, participants were asked to answer questions about sociodemographic characteristics (gender and age), year of study, and their major.

In the second part of the questionnaire, participants were asked questions related to their smartphone use. They were asked how old they were when they got their smartphone (“How old were you when you first got/bought a smartphone?”). They were also asked about the most common reasons why they use smartphones ((a) to access social networks; (b) correspondence via applications Whatsapp, Messenger, etc.; (c) make calls and send messages; (d) search for information in order to fulfil obligations at the university; (e) reading the news; or (f) other). In addition, their daily time of smartphone use and time spent on social networks was identified ((a) less than 30 min; (b) from 30 min to an hour; (c) from one to two hours; (d) from three to five hours; (e) from five to ten hours; and (f) more than ten hours), and they were asked how often they send messages and check notifications. At the end, participants were asked to answer if they use a smartphone before going to sleep (“Do you use your mobile phone before going to bed? If your answer is yes, please indicate how much time, in minutes, you spend on your mobile phone before going to bed”).

Nomophobia Questionnaire (NMP-Q) [4].

NMP-Q is a measuring instrument that provides insight into the intensity of anxiety or distress of an individual in situations when he or she cannot access a smartphone. This questionnaire consists of twenty statements divided into four factors or dimensions: *loss of connectivity* (5 statements), *inability to communicate* (6 statements), *denial of comfort* (4 statements), and *inability to access information* (4 statements).

The task of the participants is to assess the degree of agreement with individual statements on a scale of five degrees (from 1, “I completely disagree”, to 5, “I completely agree”). The total result of the participants on each subscale as well as the questionnaire as a whole is obtained by summing the estimates on the corresponding statements. In doing so, a higher score on each subscale indicates a higher level of said nomophobic factor. The author of the original version of NMP-Q determined an extremely high Cronbach’s alpha, i.e., the reliability of the internal consistency, which was 0.95 [4]. In this study, a translated version of the questionnaire was used [8], and an extremely high Cronbach alpha was confirmed, which was 0.93.

Depression, anxiety, and stress scale (DASS) [24,25].

DASS consists of 42 statements that show a three-factor structure. The scale consists of subscales of depression, anxiety, and stress, and each of the subscales includes 14 statements. The subscale of depression refers to symptoms of dysphoria, hopelessness, self-depreciation, apathy, and lack of interest. The anxiety subscale refers to the excitation of the autonomic system and certain physiological changes inherent in anxiety. On the other hand, the stress subscale includes indicators of excessive chronic, nonspecific excitation, anxiety, difficulty of relaxation, and irritability.

On all subscales, the respondent gives answers on a Likert-type scale from 0 (“did not apply to me at all”) to 3 (“totally or most of the time applied to me”). The reliability coefficient in this study is 0.95 for depression, 0.89 for anxiety, and 0.94 for stress.

The categories for the expression of individual symptoms of depression, anxiety, and stress, which are examined by the DASS scale obtained in the original research of the authors of this scale, are shown in [24].

Social and emotional loneliness scale (SELSA) [26,27].

SELSA consists of three subscales that, individually, examine loneliness in relation to the family (subscale of loneliness in the family, 11 statements) and love relationships (subscale of loneliness in love, 12 statements) and loneliness in friendly relationships (subscale of social loneliness, 13 statements).

The total result of the participants on individual subscales is obtained by summing the estimates on the corresponding statements, and a higher result on each subscale indicates greater loneliness in a given domain. In this study, the values of Cronbach’s alpha coefficient are satisfactory (social loneliness 0.86, loneliness in love 0.71, loneliness in the family 0.72).

Emotional skills and competence questionnaire (ESCQ-15) [28].

ESCQ-15 consists of 15 statements. The task of the participants is to determine their degree of agreement with each statement on a Likert-type scale, where 1 means “strongly disagree”, while 5 means “strongly agree” with the statement. The total result is obtained by summing all the above estimates. When it comes to the reliability of the *Emotional Skills and Competence Questionnaire*, it proved to be high, with a Cronbach’s alpha coefficient of 0.86.

### 2.3. Data Analysis and Statistical Methods

Data were analysed in the IBM SPSS Statistics program (version 25). The normality of distribution of data was provided. To examine the relationship between nomophobia and sociodemographic characteristics, variables associated with smartphone use, distress factors, and various emotional difficulties, Pearson’s correlation coefficients were calculated. To determine the contribution of nomophobia and emotional skills and competence in explaining the expression of distress factors (depression, anxiety, stress), a hierarchical regression analysis was performed.

## 3. Results

### 3.1. Descriptive Data of Examined Variables

Table 1 shows that the participants show moderately high levels of nomophobia (if the theoretically highest value on the *Nomophobia Questionnaire* is considered). Looking at each subscale separately, it can be concluded that the result on the subscale *loss of connectivity* is the lowest, while the results on the subscales the *inability to access information* and *denial of comfort* show a tendency to move towards higher values. The result on the subscale *inability to communicate* is quite high. It follows that the participants of this research experience a highly expressed fear of the inability to communicate via smartphones. The obtained finding should not be surprising since it is a subscale whose content largely corresponds to the definition and characteristics of nomophobia. The obtained results were confirmed in the Bodrožić Selak research, which is also the first research on nomophobia in a sample of students in Croatia [9], but in this research, there is even a slight increase in average values compared to the Bodrožić Selak research (M_1_ = 62.4; SD_1_ = 16.14 vs. M_2_ = 58.1, SD_2_ = 14.93).

Distress factors (depression, anxiety, stress), considering the theoretical range of symptoms of depression, anxiety, and stress, were moderately expressed, while the symptoms of stress were mild. Students were slightly above average in emotional skills and competencies, below-average socially lonely, and slightly below average when it comes to loneliness in the family while slightly above average in loneliness in love relative to the theoretical range results [9].

When it comes to owning a smartphone, participants obtained, on average, their first smartphone at the age of 12 (M = 12.86, SD = 2.67). Of these, the largest number of participants in the amount of 54.3% spent 3–5 h a day in front of a smartphone screen, while a slightly smaller share of participants spent 5–10 h (22.5%) and 1–2 h a day (20.2%) in front of their smartphone screen. The smallest share consists of participants who use smart devices for more than 10 h a day (1.9%) and those who use their device for 30 min to 1 h (1.2%), while none of the participants use a smart device for less than 30 min. Participants mostly use their smartphones to access social networks (Instagram, Facebook, YouTube, Pinterest) (48.1%) and correspondence via applications (WhatsApp and Messenger) (45.3%). Some of the listed reasons for using smartphones are their use for business purposes (2.7%), searching for information for fulfilling obligations at the university (1.9%), making calls and sending text messages (1.2%), and reading news (0.8%). When considering the use of social networks, the data show that 48.8% of participants spend 1 to 2 h a day on social networks, followed by 25.2% of participants who spend 3–5 h a day searching social networks, while 15.1% of them spend time on social networks for 30 min to 1 h. The smallest shares are represented by participants who spend 5–10 h on social networks (5.4%), less than 30 min (4.3%), and more than 10 h (1.2%). The frequency of checking notifications on a smartphone, on average, is 45 times a day (M = 44.41 SD = 55.18), while the range is from 3 to 200. When it comes to sending messages via smartphones, the data show that the largest number of participants send 0–50 messages (34.4%) per day, and a slightly smaller share of participants, more precisely (22.2%) send 50–100 messages per day. Further, 12% of participants send 100–150 messages a day, and the smallest percentage of participants send over 200 messages a day (4.7%). Finally, almost all participants use smart devices before going to bed (97.8%), of which the largest share (25.1%) spends 16 to 30 min in front of a smart device screen, 14.2% of participants spend 31 to 45 min using a smart device, while 11.2% of them use a smartphone more than 1 h before bedtime. The share of those who use the benefits of smartphones from 46 to 60 min before going to bed is slightly lower (10.3%). Of concern is the fact that the smallest share, only 9.5% of participants, spends less than 15 min on their smart devices before going to bed.

### 3.2. Correlation between Nomophobia and Other Examined Variables in Research

To examine the relationship between nomophobia and sociodemographic characteristics (gender and age), variables associated with smartphone use (age of receiving the first smartphone, average daily smartphone use, average smartphone use before bedtime, and average time spent on social media, frequency of checking notifications, and a daily number of messages sent), distress factors (depression, anxiety, stress), and various emotional difficulties (emotional skills and competence and emotional and social loneliness) along with Pearson’s and Spearman’s coefficients of correlation were calculated (Table 2). Spearman’s coefficient of correlation was used for items related to the smartphone use.

Table 2 shows that significant correlations were obtained between sociodemographic characteristics and nomophobia. Specifically, female students and younger participants experience more intense symptoms of nomophobia to a greater extent. Furthermore, a significant negative correlation was found between nomophobia and the age of receiving the first smartphone, which indicates that those individuals who own a smartphone from an earlier age experience higher level of nomophobia. In addition, individuals who use a smartphone more and spend more time on social media, send more messages, check notifications more often, and those who spend more time on a smartphone before bedtime report more intense symptoms of nomophobia. When it comes to the relationship between distress factors (depression, anxiety, stress) and nomophobia, significant, moderate correlations between these variables were found, which indicates that individuals with a greater fear of not being able to use smartphones also report more intense symptoms of depression, anxiety, and stress. At last, a significant negative association between emotional skills and competencies and nomophobia was found, and individuals with poorer emotional skills and competencies reported more intense symptoms of nomophobia, while no significant association was found between social loneliness, loneliness in love, loneliness in the family, and nomophobia.

### 3.3. Correlation between Distress Factors (Depression, Anxiety, and Stress) and Other Examined Variables

To examine the relationship between distress factors and sociodemographic characteristics (gender and age), variables associated with smartphone use (age of receiving the first smartphone, average daily smartphone use, average smartphone use before bedtime, and average time spent on social media, frequency of checking notifications, and a daily number of messages sent), nomophobia, and emotional skills and competence along with Pearson’s and Spearman’s coefficients of correlation were calculated (Table 3).

Significant correlations were obtained between all measures of distress factors (depression, anxiety, and stress), nomophobia, and emotional skills. Students who reported more intense of nomophobia have more intense symptoms of depression, anxiety, and stress, and students who have poorer emotional skills and competencies reported more intense symptoms of depression, anxiety, and stress. Only the variable “average time spent on social media” showed a positive correlation with all distress factors. Students who spent more time on social networks reported more depressive, anxiety, and stress symptoms. There was no significant relationship between age and distress factors, and there was no significant relationship between gender and depression. It was found that gender has a significant relationship with anxiety and stress, and females experience more intense symptoms.

### 3.4. The Role of Age, Gender, Nomophobia, and Emotional Skills and Competence in Explaining Distress Factors

To determine the contribution of nomophobia and emotional skills and competence in explaining the expression of distress factors (depression, anxiety, stress), a hierarchical regression analysis was performed. There is not enough research on the study of nomophobia and how the daily use of smartphones affects mental health because it is a relatively new concept; thus, we decided to include in the regression analysis those factors that are related to distress factors, based on Table 3.

In the first step of the hierarchical regression analysis, the variable “age” was introduced. Table 4, Table 5 and Table 6 show that the variable “age” did not significantly contribute to explaining the symptoms of depression, anxiety, and stress. In the second step, when variable of “gender” was introduced, it was shown that gender did not significantly contribute to explaining the symptoms of depression but did contribute to explaining the symptoms of anxiety and stress. By introducing gender in the second step of the analysis, the percentage of explained variance of anxiety increased by 3.4%, while the percentage of explained variance of stress increased by 2.5%. This set of predictors (age and gender) in anxiety explain 4.4% of symptomatology and 2.7% of symptoms of stress.

In the third step, the variable “average time spent on social networks” was introduced, and it significantly contributed to explaining the symptoms of depression, anxiety, and stress, and gender remained significant in the explanation of anxiety and stress. The results suggest that students who spent more time on social networks experience more intense symptoms of depression, anxiety, and stress, and especially female students, who spent more time on social networks, have more intense symptoms of anxiety and stress. By introducing average time spent on social networks in the third step of analysis, the percentage of explained variance of depression symptoms increased by 2.4%, and the percentage of explained variance of anxiety symptoms increased by 2.2% and by 1.7% for stress symptoms. The third set of predictors in depression and stress explains 4% symptomatology and in anxiety, 6.6%.

In the fourth step, when the variable of nomophobia was introduced, previous predictors (age, gender, average time spent on social network) became insignificant, and they no longer significantly contributed to explaining the variance in the severity of depression, anxiety, and stress. While nomophobia has proven to be a significant and strong predictor in explaining the severity of symptoms of depression, anxiety, and stress, the result suggests that individuals who report higher levels of nomophobia also experience more intense symptoms of depression, anxiety, and stress. By introducing nomophobia in the fourth step of the analysis, the percentage of explained variance of depression symptoms increased by 8.5%, while the percentage of explained variance of anxiety increased by 13.4%, and stress symptoms increased by 14.5%; so now, this set of predictors in depression explains 12.4% of symptomatology and 20% and 18.9% of symptoms of anxiety and stress, respectively.

In the fifth and last step of hierarchical regression analysis, the variable of emotional skills and competence was introduced. In this step, “average time spent on social networks” still does not significantly contribute to explaining the variance in the severity of symptoms of depression, anxiety, and stress, while nomophobia still retains the status of a significant predictor. In addition to nomophobia, emotional skills and competencies are a significant negative predictor of the severity of symptoms of depression, anxiety, and stress. Specifically, individuals who report poorer emotional skills and competencies experience more intense symptomatology of depression, anxiety, and stress. The introduction of emotional skills and competence in the hierarchical regression analysis additionally explained 8.3% of the variance of anxiety symptoms, 6.7% of the variance of stress symptoms, and 18.6% of the variance of depression symptoms. Only in depression, emotional skills and competencies proved to be a better predictor than nomophobia. At last, the final step of the hierarchical regression analysis explained 31% of the variance in the severity of depression symptoms, 23.8% of the variance in the severity of anxiety symptoms, and 25.6% of the variance in the severity of stress symptoms.

## 4. Discussion

Smartphones and smart watches have become an integral part of everyday life. Mobile devices are considered as perhaps the biggest “addiction” of the 21st century [29]. The daily overuse of smart technologies, which in most people exceeds 9 h a day, is fertile ground for the development of addiction. The term “technology paradox” is often used to refer to the fact that technology has the function of both liberation and enslavement. While it provides a relief from the real world, it also often introduces its users to the slavery of the virtual world. Although smartphones have made everyday life much easier, the negative effects of their problematic use have been rapidly increasing in recent years. Excessive use of mobile devices can negatively affect academic performance and success of individuals. It can also cause interpersonal relationships, sleep difficulties, and various mental disorders [30]. Recently, research on the relationship between mental health and the use of modern technologies has given rise to a new concept worthy of attention—nomophobia. It involves experiencing anxiety in situations where a person cannot access a smartphone and the tools it provides. Since this is a new phenomenon, there is not much research on this topic. However, the number of studies that seek to gain better understanding of this new phenomenon is growing every day. Due to the era in which we live and its rapid technological progress, the prevalence of nomophobia is increasing. Hence, in the future, we can expect a higher percentage of people with severe symptoms of nomophobia.

There is positive correlation between the levels of nomophobia and the gender variable; more precisely in this research, women report a more pronounced fear of not being able to use smartphones, and this was confirmed in some previous studies [31,32]. One possible explanation for this result is the fact that women, on average, spend more time using their mobile device, and nomophobia is associated with excessive use of smart devices. Women compared to men are more likely to use smartphones for socialization purposes and experience higher levels of social stress [33]. Furthermore, women, unlike men, talk more through their mobile devices. Thus, women are more likely to develop some form of mobile addiction due to social stress and the way they use smartphones [33].

Research showed that younger individuals report higher levels of nomophobia, and a negative association between the age variable and the level of nomophobia was found. If we look at the connections at the level of individual dimensions of nomophobia, we can conclude that younger individuals express a more intense fear of not being able to communicate via smartphone. This observation is not surprising since it is the dimension that, in theoretical terms, best fits the construct of nomophobia. The findings are confirmed by the research, which found that the levels of nomophobia and the severity of its symptoms decrease with age [34]. According to them, younger individuals have lower impulse control when using smartphones. In addition, smartphones represent status symbol for them.

When looking at the relationship between nomophobia and different ways of using smartphones, it is assumed that variables related to the use of smartphones, such as time spent on social networks, average daily smartphone use, daily number of sent messages and frequency of notifications, and time spent on smart device before going to bed, are positively correlated with nomophobia. Individuals who, on average, spend more time on social media, send more messages, check mobile phone notifications more often, spend more time on a mobile device before bed, and those who spend more time globally using their own mobile device experience more intense fear of the potential impossibility of the use of the same. A possible explanation for this is the behavioural approach that interprets various everyday patterns of behaviour of an individual related to the use of smart devices, such as searching social networks, as routine actions that have become so routine that they resemble automatic actions, and situations that do not allow performing certain behaviours, such as searching social networks, activates processes that underlie the experience of anxiety or nomophobia [9]. Finally, it was assumed that the age of possession of the first smart device would be negatively associated with levels of nomophobia, and our findings confirms this assumption. Specifically, individuals who own a smartphone from an early age report higher levels of nomophobia. This finding is consistent with research that found that younger individuals are a risk group for developing nomophobia due to poorer self-regulatory abilities [33].

This study found moderate positive correlations between distress factors and the average score on the Nomophobia Questionnaire, which is in line with the results of previous research [35]. Namely, it was suggested that excessive use of smart devices often precedes the development of anxiety disorders, and the absence of these devices intensifies distress [35]. Furthermore, since many researchers consider nomophobia to be a specific phobia belonging to the group of anxiety disorders, the correlation between anxiety and nomophobia is not surprising [36]. Furthermore, it was found that those who reported experiencing higher levels of stress also reported higher levels of nomophobia. These findings are in line with previous research that considers social networks to be a chaotic environment due to limited control over their existence in the cyber world, and this results in stress [37]. An additional explanation of the obtained connection stems from the theory of compensatory use, according to which users change the level of Internet use in accordance with life problems [38]. Specifically, when faced with various problems, individuals resort to frequent use of the Internet, aiming at managing their own mood.

In addition to stress, individuals with more pronounced depressive symptomatology have been shown to report higher levels of nomophobia, which is in line with our expectations. The link between nomophobia and stress can be explained in the context of escape. Namely, depressed individuals “run” to the Internet to suppress all the negative emotions that overwhelm them, and many studies show that, unfortunately, the opposite is happening. Namely, social networks are a great platform for creating a false image of yourself. Many people, in order to gain the approval of the environment, share unrealistic images of their lifestyles, travels, partners, bodies, and food. The problem arises because individuals receive the impression that the lives of others are better than their own, which further contributes to negative emotions and intensifies depressive symptoms instead of leading to their reduction [13]. This is confirmed by the research that explained envy as a mechanism that connects the use of Facebook with depression, while the use of the social network itself does not lead to the strengthening of depression [39].

This study found that people with lower emotional competencies and skills report higher levels of nomophobia. This is in line with previous research showing that lower emotions regulation and management skills and hence lower emotional competencies and skills predict nomophobia traits [40]. More precisely, technology and above all social networks provide “emotionally fragile” people who have a problem in everyday, face-to-face communication with the opportunity to easily slip into a new, false identity that they have tailored. They become dependent on their means of escape and thus feel great discomfort and anxiety if they are unable to access their own smartphone [41]. People who suffer from an anxiety disorder when they find themselves in stressful and anxious situations use their smart devices as a source of security because of low emotion regulation [14]. Finally, smartphones can serve as a means of escaping real-world problems for individuals who have emotional difficulties [42,43].

Hierarchical regression analysis found that people who spend a long time on social networks experience higher levels of depression, anxiety, and stress, i.e., report more pronounced symptoms. This is supported by the research of Franco and Carrier, which found that increased social network searching increases stress and anxiety [44]. Previous research found a positive association between the use of social networks and the severity of depression, anxiety, and stress; i.e., social networks were a significant predictor of depression, anxiety, and stress [45]. Furthermore, individuals who experience a more intense fear of not being able to use smart devices also report more pronounced depressive and anxiety symptoms and more stress [46]. It is an indisputable fact that nomophobia, as a modern form of pathology, plays an important role in the mental health and behavioural changes of an individual. Research has shown that people who have poorer emotional skills and competencies experience more pronounced current symptoms of depression, anxiety, and stress. It is also important to mention that emotional skills and competencies are the strongest predictor in the case when the criterion was depression. Starting from the thesis that emotional difficulties are behind a wide range of psychopathological phenomena and disorders and especially mood disorders, the result is not surprising.

Even though this research offers some new insights into nomophobia, which is a relatively new construct evolving in parallel with advances in technology and its relationship to variables that are extremely important for an individual’s daily functioning, it is important to point out some limitations of this research. Primarily, the research does not provide information on potential cause-and-effect relationships between variables, as it is fundamentally correlative in nature, but only provides information on the existence of consistency in their variation. Therefore, it would be very useful to conduct a longitudinal study that can capture and monitor changes in the variables examined in this study and draw a conclusion about possible cause-and-effect relationships. In addition, since we analysed data, we found that students spend a great deal of time behind the screen of their mobile device, and numerous studies indicate a negative impact of mobile devices on sleep quality [47,48,49]. In addition, research indicates the negative impact of insomnia on emotional regulation and consequently on mental health [50]. Hence, future research should include the sleep variable in the study. Moreover, in this study, there is large difference in the distribution of the sample according to sex. Our sample consisted of mainly women because literature shows that nomophobia rates are much higher in women [51,52]. However, future research should further explore sex differences.

## 5. Conclusions

In our study, younger individuals and women reported higher levels of nomophobia. Furthermore, individuals who own a smart device from an early age reported higher levels of fear of not being able to use a smart device. In addition, several hours of smartphone use, frequent checking of messages and notifications, long periods of time on social networks, as well as prolonged use of smart devices before bedtime were all associated with higher levels of nomophobia. In conclusion, individuals with more pronounced symptoms of nomophobia reported more pronounced symptoms of depression, anxiety, and stress as well as lower emotional skills and competencies.

By looking at the individual relative contributions of emotional skills and competencies and nomophobia in explanation of the symptom severity of depression, anxiety, and stress, it was found that all variables significantly contribute to explaining the symptoms of depression, anxiety, and stress. In other words, people with lower emotional skills and competencies and a more intense fear of not being able to use smart devices reported more pronounced symptoms of depression, anxiety, and stress. In addition, the average time spent on social media has appeared to be a significant predictor of symptom severity of depression, anxiety, and stress. This means that individuals who spend a great deal of time on social media report more pronounced symptoms of depression, anxiety, and stress.

## Figures and Tables

**Table 1:** Descriptive data for the measured variables (N = 257).

Variables	M	SD	Min	Max
***NMP-Q***				
Inability to access information	12.60	3.98	4	20
Denial of comfort	16.28	4.38	6	25
Inability to communicate	21.84	5.81	6	30
Loss of connectivity	11.68	4.70	5	25
Nomophobia (total score)	62.40	16.14	23	100
**DASS**	13.49	11.26	0	42
Depression				
Anxiety	11.17	8.84	0	40
Stress	17.27	11.16	0	42
***ESCQ-15***	55.15	9.52	15	74
Emotional skills and competence				
***SELSA***				
Social loneliness	30.53	12.89	13	81
Loneliness in love	43.46	7.06	24	71
Loneliness in family	34.42	10.19	15	64

**Table 2:** Correlation between nomophobia, individual measures of nomophobia, and other examined variables (N = 257).

Variables	Inability to Access Information	Denial of Comfort	Inability to Communicate	Loss of Connectivity	Nomophobia Overall
Gender	0.14 *	0.22 **	0.30 **	0.14 *	0.25 **
Age	−0.10	−0.20 **	−0.15 *	−0.10	−0.14 *
Age of receiving the first smartphone	−0.16 **	−0.17 **	−0.17 **	−0.11	−0.15 **
Average daily smartphone use	0.30 **	0.31 **	0.23 **	0.26 **	0.31 **
Average time spent on social networks	0.29 **	0.28 **	0.15 **	0.38 **	0.30 **
Daily number of messages sent	0.12	0.12	0.15 *	0.07	0.14 *
Frequency of checking the notification	0.19 **	0.31 **	0.19 **	0.16 **	0.25 **
Average smartphone use before sleep	0.25 **	0.22 **	0.16 **	0.23 **	0.24 **
***ESCQ-15***					
Emotional skills and competence	−0.14 *	−0.26 **	−0.01	−0.12	0.10
***SELSA*** Social loneliness	0.01	0.14 *	−0.09	0.01	0.02
Loneliness in love	0.03	0.05	−0.10	0.03	−0.01
Loneliness in family	0.06	0.22 **	−0.06	0.15 *	0.09
***DASS***					
Depression	0.30 **	0.38 **	0.26 **	0.31 **	0.35 **
Anxiety	0.38 **	0.46 **	0.31 **	0.39 **	0.44 **
Stress	0.38 **	0.46 **	0.31 **	0.37 **	0.440 **

Note: * *p* < 0.05; ** *p* < 0.01.

**Table 3:** Relationship between distress factors and sociodemographic characteristics, smartphone use, nomophobia and emotional skills and competence (N = 257).

	Depression	Anxiety	Stress
Age	−0.05	−0.10	−0.05
Gender	−0.11	−0.18 **	−0.16 **
Average daily smartphone use	0.11	0.09	0.12
Average time spent on social network	0.20 **	0.18 **	0.16 **
Frequency of notification checking	−0.04	−0.01	0.01
Daily number of messages sent	0.11	−0.01	0.06
Average smartphone use before bedtime	0.09	0.06	0.12
Nomophobia	0.35 **	0.44 **	0.44 **
Emotional skills and competence	−0.47 **	−0.38 **	−0.31 **

Note: ** *p* < 0.01.

**Table 4:** Hierarchical regression analysis in predicting depression (N = 257).

Depression							
						Collinearity Statistics	
**Predictors**	***1. Step***	***2. Step***	***3. Step***	***4. Step***	***5. Step***	**Tolerance**	**VIF**
	β	β	β	Β	β		
Age	−0.049	−0.053	−0.031	0.006	0.011	0.934	1.071
Gender		−0.116	−0.104	−0.037	−0.082	0.964	1.038
Average time spent on social networks			−156 *	0.053	0.034	0.868	1.152
Nomophobia				0.321 **	0.260 **	0.809	1.236
Social skills and competence					−0.438 **	0.791	1.030
**R**	0.049	0.126	0.199	0.352	0.557		
**R^2^**	0.002	0.016	0.040	0.124	0.310		
**R^2^ Adj**	−0.001	0.008	0.028	0.110	0.296		
**ΔR^2^**	0.002	0.013	0.024 *	0.084 **	0.186 **		

Note: * *p* < 0.05; ** *p* < 0.01; dependent variable: depression; VI, variance inflation factor.

**Table 5:** Hierarchical regression analysis in predicting anxiety in (N = 257).

Anxiety							
						Collinearity Statistics	
**Predictors**	**1. Step**	**2. Step**	**3. Step**	**4. Step**	**5. Step**	**Tolerance**	**VIF**
	*β*	*β*	*β*	*β*	*β*		
Age	−0.099	−0.104	−0.083	−0.037	−0.033	0.934	1.071
Gender		−0.184 **	−0.173 **	−0.089	−0.118	0.964	1.038
Average time spent on social networks			0.151 **	0.022	0.009	0.868	1.152
Nomophobia				0.403 **	0.363 **	0.809	1.236
Social skills and competence					−0.293 **	0.791	1.030
**R**	0.099	0.209	0.257	0.447	0.532		
**R^2^**	0.010	0.044	0.066	0.200	0.238		
**R^2^ Adj**	0.006	0.036	0.055	0.187	0.269		
**ΔR^2^**	0.010	0.034 **	0.022 **	0.134 **	0.083 **		

Note: ** *p* < 0.01; dependent variable: anxiety; VIF, variance inflation factor.

**Table 6:** Hierarchical regression analysis in predicting stress (N = 257).

Stress							
						Collinearity Statistics	
**Predictors**	**1. Step**	**2. Step**	**3. Step**	**4. Step**	**5. Step**	**Tolerance**	**VIF**
	*β*	*β*	*β*	*β*	*β*		
Age	−0.039	−0.044	−0.026	0.023	0.026	0.934	1.071
Gender		−0.160 **	−0.150 *	−0.062	−0.089	0.964	1.038
Average time spent on social networks			−0.132 *	−0.003	−0.014	0.868	1.152
Nomophobia				0.420 **	0.384 **	0.809	1.236
Social skills and competence					−0.263 **	0.791	1.030
**R**	0.039	0.164	0.210	0.435	0.506		
**R^2^**	0.002	0.027	0.044	0.189	0.256		
**R^2^ Adj**	−0.002	0.019	0.033	0.176	0.241		
**ΔR^2^**	0.002	0.025 **	0.017 *	0.145 **	0.067 **		

Note: * *p* < 0.05; ** *p* < 0.01; dependent variable: stress; VIF, variance inflation factor.

## Data Availability

The data supporting the conclusions of this article will be made available by the authors, without undue reservation.
